# In vitro cytotoxicity and antibacterial activity of silver-coated electrospun polycaprolactone/gelatine nanofibrous scaffolds

**DOI:** 10.1007/s13205-016-0531-6

**Published:** 2016-09-29

**Authors:** Mim Mim Lim, Naznin Sultana

**Affiliations:** 1 Department of Clinical Sciences, Faculty of Biosciences and Medical Engineering, Universiti Teknologi Malaysia, Johor Bahru, 81310 Johor, Malaysia; 2 Advanced Membrane Technology research Center (AMTEC), Universiti Teknologi Malaysia, Johor Bahru, 81310 Johor, Malaysia

**Keywords:** Polycaprolactone (PCL), Gelatine (Ge), Silver (Ag), Nanofibrous scaffold, Antibacterial property, Cytotoxicity

## Abstract

The development of nano-sized scaffolds with antibacterial properties that mimic the architecture of tissue is one of the challenges in tissue engineering. In this study, polycaprolactone (PCL) and PCL/gelatine (Ge) (70:30) nanofibrous scaffolds were fabricated using a less toxic and common solvent, formic acid and an electrospinning technique. Nanofibrous scaffolds were coated with silver (Ag) in different concentrations of silver nitrate (AgNO_3_) aqueous solution (1.25, 2.5, 5, and 10 %) by using dipping method, drying and followed by ultraviolet (UV) photoreduction. The PCL/Ge (70:30) nanofibrous scaffold had an average fibre diameter of 155.60 ± 41.13 nm. Characterization showed that Ag was physically entrapped in both the PCL and PCL/Ge (70:30) nanofibrous scaffolds. Ag^+^ ions release study was performed and showed much lesser release amount than the maximum toxic concentration of Ag^+^ ions in human cells. Both scaffolds were non-toxic to cells and demonstrated antibacterial effects towards Gram-positive *Bacillus cereus* (*B. cereus*) and Gram-negative *Escherichia coli* (*E. coli*). The Ag/PCL/Ge (70:30) nanofibrous scaffold has potential for tissue engineering as it can protect wounds from bacterial infection and promote tissue regeneration.

## Background

Tissue engineering has emerged to provide a new medical therapy in helping tissue regrowth and regeneration (Sultana 2003; Groeber et al. 2011). It employs a scaffold as an artificial supporting structure for cellular growth. There are many techniques in scaffold fabrication, including electrospinning (Hassan et al. 2014; Yu et al. 2013; Zhu et al. 2012). The electrospinning technique was used in this research as it produces a fibrous scaffold that mimics the natural extracellular matrix (ECM) of the dermis. This technique uses an electric field to convert a polymer solution into fibres by creating a charged jet of polymer solution that is ejected and travels in the air to form micro- and nano-sized fibres (Rujitanaroj et al. 2008).

To fabricate a scaffold, biomaterials such as biodegradable polymers are widely used as the material of the scaffold. Using a biodegradable polymer as the scaffold provides adequate support for cells and degrades at a rate coincident with tissue growth. Biodegradable polymers are divided into biodegradable synthetic polymers and natural polymers. In this study, a biodegradable synthetic polymer, polycaprolactone (PCL), and a natural polymer, gelatine (Ge), were used. Both of these polymers have been approved by the Food and Drug Administration (FDA) and are safe for use in the human body (Kiran et al. 2014; GMIA 2012). Some studies have observed that PCL fibrous scaffolds cause a reduction in cell attachment, migration, proliferation, and differentiation (Jin et al. 2015). Cell affinity toward synthetic polymers is usually poorer than toward natural polymers. PCL has the advantages of biodegradability and biocompatibility, but it has lower hydrophilicity, a slower degradation rate, and lacks surface cell-recognition sites (Chong et al. 2015). Ge is a natural polymer derived from collagen, the main structural component of the ECM of skin. It is hydrophilic and has a faster degradation rate. Due to the merits of its biological origin, it was selected to be blended with PCL to achieve the desired fibrous scaffold to mimic the ECM with enhanced wettability, a faster degradation rate, and improved cell attachment as well as proliferation.

Although the choice of biomaterial is crucial to wound healing, bacterial colonization and infection can also affect the healing process of deep dermal injuries. Some studies have been conducted to incorporate antibiotic or antibacterial agents to inhibit the growth of bacteria. Incorporation of a hydrophilic antibiotic (Mefoxin^®^, cefoxitin sodium) into poly(lactide-*co*-glycolide) (PLGA) nanofibrous scaffolds was found to inhibit the growth of *Staphylococcus aureus* (*S. aureus*) (Kim et al. 2004). Electrospun Ge fibres containing silver (Ag) nanoparticles have shown antibacterial activity against *Pseudomonas aeruginosa*, *S. aureus*, *Escherichia coli* (*E. coli*), and methicillin-resistant *S. aureus* (Rujitanaroj et al. 2008). It has also been reported that metal ions such as zinc, copper, and Ag have strong antibacterial activity (Li et al. 2007; Ibrahim et al. 2011; Depan et al. 2011). Ag is the most well-known metallic antibacterial agent and has been used in various biomedical applications. The maximum toxic concentration of Ag for human cells is 10 mg/L (Vik et al. 1985). High concentrations of Ag may cause cytotoxicity, but low concentrations of Ag are non-toxic and safe for medical use (Stanić et al. 2011; Alt et al. 2004; Xing et al. 2010).

In this study, PCL and PCL/Ge (70:30) fibrous scaffolds were fabricated using the electrospinning technique. These scaffolds were not incorporated with an antibacterial agent, which might cause bacterial infection and inflammation in the wound. Hence, integration of an antibacterial agent on scaffold is worth investigating. Ag was coated on the PCL and PCL/Ge (70:30) fibrous scaffolds and tested for in vitro cell cytotoxicity on human skin fibroblasts (HSF) as well as antibacterial activities using bacteria that cause skin and wound infections: *Bacillus cereus* (*B. cereu*s) and *E. coli* (Bottone 2010; John et al. 2012; Rennie et al. 2000; Church et al. 2006). These tests were done to assess the possibility of using these silver entrapped scaffolds for skin tissue engineering applications.

## Materials and methods

### Materials

PCL (molecular weight 70,000–90,000), Ge powder (type A; porcine skin; ~300 g Bloom), phosphate buffered saline (PBS, P-3813, pH 7.4), and formic acid (≥95 %) were supplied from Sigma-Aldrich. Silver nitrate (AgNO_3_, Grade AR, 169.87 g/mol) and dimethyl sulphoxide (DMSO, Grade AR) were supplied from QReC. 3-(4,5-dimethylthiazol-2-yl)-2,5-Diphenyltetrazolium bromide (MTT, M6494) was purchased from Molecular Probes by Life Technologies and Dulbecco’s Modified Eagle Medium (DMEM, high glucose, 12,100–038), was purchased from Gibco by Life Technologies. HSF (1184), *E. coli* (25,922), and *B. cereus* (13,061) were supplied by the American Type Culture Collection (ATCC).

### Fabrication and characterization of PCL and PCL/Ge (70:30) nanofibrous scaffolds

#### Preparation of PCL and the PCL/Ge (70:30) solution

14 % w/v PCL and PCL/Ge solutions with a weight ratio of 70:30 were prepared by dissolving PCL and Ge in formic acid and stirring for 3 h at room temperature using a magnetic stirrer.

#### Electrospinning of PCL and PCL/Ge (70:30) nanofibrous scaffolds

The electrospinning process was conducted by using a NaBond Nanofiber Electrospinning Unit (China). The PCL solution was loaded into a syringe with a 23 gauge stainless steel needle and connected to a high-voltage supply. By using a syringe pump (NE-300, New Era Pump Systems, Inc.), the PCL solution was forced through the needle at a controlled rate, i.e., 1 ml/h. A high voltage of 17 kV was applied for 2 h at a distance of 10 cm between the capillary tip and the collector. Fibres were formed and deposited onto aluminium foil as a grounded collector.

#### Characterization

PCL and PCL/Ge (70:30) nanofibrous scaffolds were sputter-coated with gold and viewed under field emission scanning electron microscope (FESEM, SU8020, Hitachi). Image J software was used to measure the fibre diameter and pore size. Average fibre diameters and pore sizes were calculated by taking the average of 20 measurements. All fibre diameters and pore sizes are presented as mean ± standard deviation (SD).

### Ag/PCL and Ag/PCL/Ge (70:30) nanofibrous scaffolds

#### Ag coating

In order to coat the nanofibrous scaffolds, dipping method in AgNO_3_ aqueous solution was used in this study. AgNO_3_ aqueous solution was prepared by dissolving AgNO_3_ in distilled water and stirred using magnetic stirrer. To coat the PCL and PCL/Ge (70:30) nanofibrous scaffolds with Ag, the samples were immersed in a 1.25, 2.5, 5, or 10 % w/v silver nitrate (AgNO_3_) aqueous solution for 1 hour. After an hour of immersion, both scaffolds were allowed to dry in a desiccator at room temperature and followed by UV photoreduction for 1 h. Colour changes of Ag-coated PCL and PCL/Ge (70:30) nanofibrous scaffolds were observed.

#### Characterization

The Ag distribution on nanofibrous scaffolds was observed using energy-dispersive X-ray spectroscopy (EDX) (TM3000 Tabletop, Hitachi), EDX mapping, and the weight percentage of Ag was observed using EDX. Chemical bonding analysis of Ag/PCL and Ag/PCL/Ge (70:30) nanofibrous scaffolds were performed by attenuated total reflectance (ATR) spectroscopy in the range of 4000–400 cm^−1^. Spectra were analysed using IR solution software.

#### Ag^+^ ion release study

Ag/PCL and Ag/PCL/Ge (70:30) nanofibrous scaffolds were sectioned into 1 × 1 cm pieces and placed in a Falcon tube containing 10 ml of PBS (pH 7.4). These scaffolds were put in a water bath at 37 °C for 1, 3, and 7 days. After 1, 3, and 7 days, the concentration of silver in the PBS was determined by atomic absorption spectroscopy (AAS) (PerkinElmer AAnalyst 400) (Xing et al. 2010; Xu et al. 2006).

#### Antibacterial evaluation

The antibacterial activity of PCL, PCL/Ge (70:30), Ag/PCL, and Ag/PCL/Ge (70:30) nanofibrous scaffolds were investigated by the zone of inhibition method. Nanofibrous scaffolds were cut into circular discs (1.4 cm in diameter). An Ag disc of 1.2 cm in diameter was used as the positive control by using *E. coli* (Gram-negative bacteria) and *B. cereus* (Gram-positive bacteria) as the model microorganisms. By using the spread plate method, a nutrient agar plate was inoculated with 1 ml of a bacterial suspension containing around 10^8^ cfu/ml of each bacteria. Scaffolds were gently placed on the inoculated plates and incubated at 37 °C for 24 h. Zones of inhibition were determined by measuring the clear area that formed around each scaffold.

#### Cell cytotoxicity using the MTT assay

The MTT assay was used to test for cell cytotoxicity on Ag-coated PCL and PCL/Ge (70:30) nanofibrous scaffolds with 10 % w/v AgNO_3_. HSF cells cultured in wells without the scaffold were used as the control. Nanofibrous scaffolds were sectioned into circular discs (1.6 cm in diameter) and sterilized by washing three times using PBS with 1 % penicillin/streptomycin followed by exposing the pieces to UV radiation for 2 h. 10 × 10^3^ cells/well were seeded on the scaffolds for 3 days in 24-well plates with DMEM. After 3 days, the DMEM was removed. New DMEM and MTT solution (5 g/l) were added to each well followed by incubation for 4 h. The plates were wrapped with aluminium foil as MTT is sensitive to light. After 4 h, purple formazan was formed. The medium was removed and 1 ml of DMSO was added to dissolve the formazan. After all formazan had dissolved, a 100 µl aliquot was transferred to a 96 well-plate, with five replicates for each sample. DMSO was set as the blank. Absorbance was measured at a wavelength of 570 nm using a microplate spectrophotometer (Epoch, BioTek).

### Statistical analysis

All data are expressed as mean values ± SD. Statistical analyses were carried out using ANOVA. A *p* value <0.05 was considered statistically significant.

## Results and discussion

### Fabrication and characterization of PCL and PCL/Ge (70:30) nanofibrous scaffolds

**Fig. 1:**
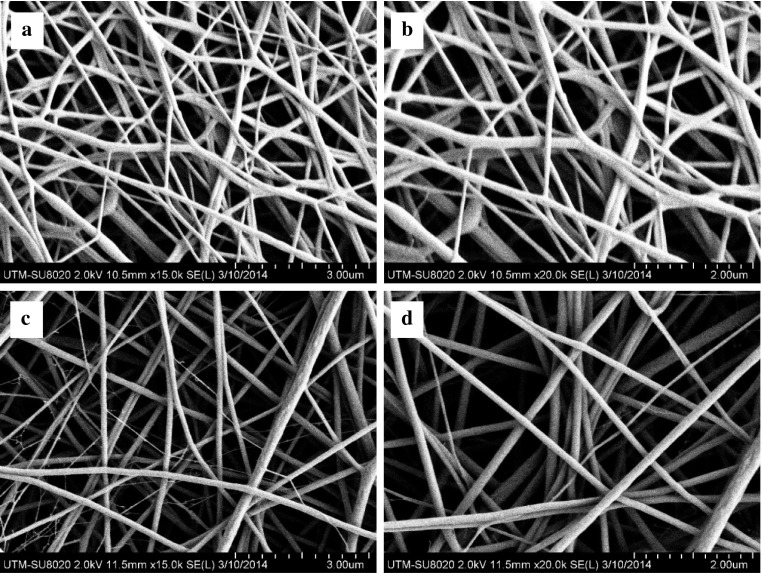
FESEM images of nanofibrous scaffolds: **a**, **b** PCL and **c**, **d** PCL/Ge (70:30)

PCL and PCL/Ge (70:30) nanofibrous scaffolds were successfully fabricated using the electrospinning technique. Figure 1 shows the morphology of these nanofibrous scaffolds. By using the parameters of voltage 17 kV, flow rate 1 ml/h, and a distance between the capillary tip and collector of 10 cm, continuous and non-beaded fibres were formed.

Average fibre diameter of the PCL and PCL/Ge (70:30) nanofibrous scaffolds was 88.02 ± 27.83 nm and 155.60 ± 41.13 nm, respectively. All scaffolds were nano-sized. The addition of Ge increased the average fibre diameter of the PCL nanofibrous scaffold. The average fibre diameter of PCL/Ge (70:30) was in the range of the collagen fibre diameter in ECM, which is 100–500 nm (Shimizu 2007). By mimicking the ECM, PCL/Ge (70:30) nanofibrous scaffold should provide a similar environment for cell growth.

The average pore size of the PCL and PCL/Ge (70:30) nanofibrous scaffolds was 811.38 ± 343.42 nm and 802.52 ± 351.37 nm, respectively. The average pore size of both nanofibrous scaffolds was similar and the scaffolds were highly porous. Pores are important for nutrient and waste product transportation, gaseous exchange (Bhardwaj and Kundu 2010) and to promote cell migration (Pham et al. 2006). A small pore size can act as a good barrier to the skin and prevent bacterial penetration.

### Ag/PCL and Ag/PCL/Ge (70:30) nanofibrous scaffolds

**Fig. 2:**
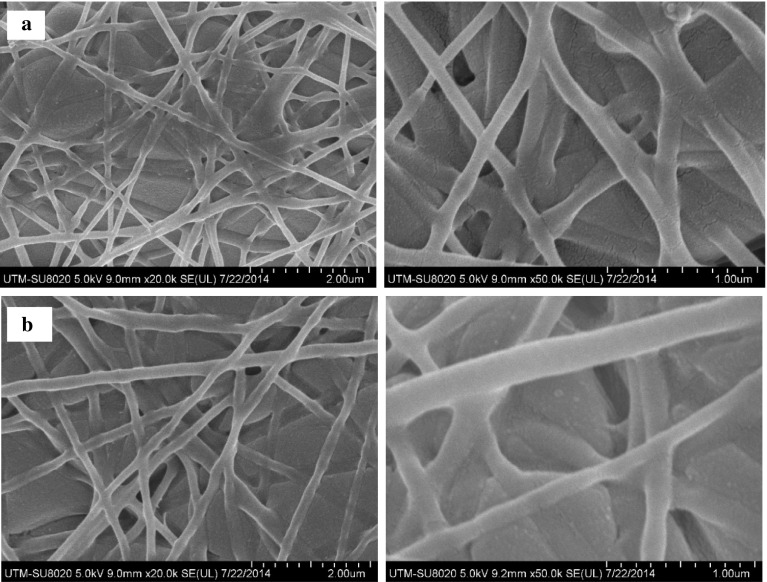
FESEM images of Ag-coated nanofibrous scaffolds with 20000× magnification (*left*) and 50000× magnification (*right*). **a** Ag/PCL and **b** Ag/PCL/Ge

PCL and PCL/Ge (70:30) nanofibrous scaffolds do not have antibacterial properties, which may lead to bacterial infections. To prevent this, scaffolds with an antibacterial effect are crucial for skin tissue engineering. Ag has been used as a promising antibacterial agent for centuries. It is effective against aerobic and anaerobic bacteria, fungi, and viruses (Jeong et al. 2014). In this study, Ag was selected as the antibacterial agent to be coated on the surface of the PCL and PCL/Ge (70:30) nanofibrous scaffolds. Figure 2 shows the FESEM images of Ag-coated nanofibrous scaffolds. Different weight percentages of Ag were coated and characterized in terms of Ag distribution, weight percentage, and chemical bonding, and the materials were studied for Ag^+^ ion release. The cytotoxicity of Ag-coated nanofibrous scaffolds towards HSF cells and their antibacterial properties were also investigated.

#### Ag coating

**Fig. 3:**
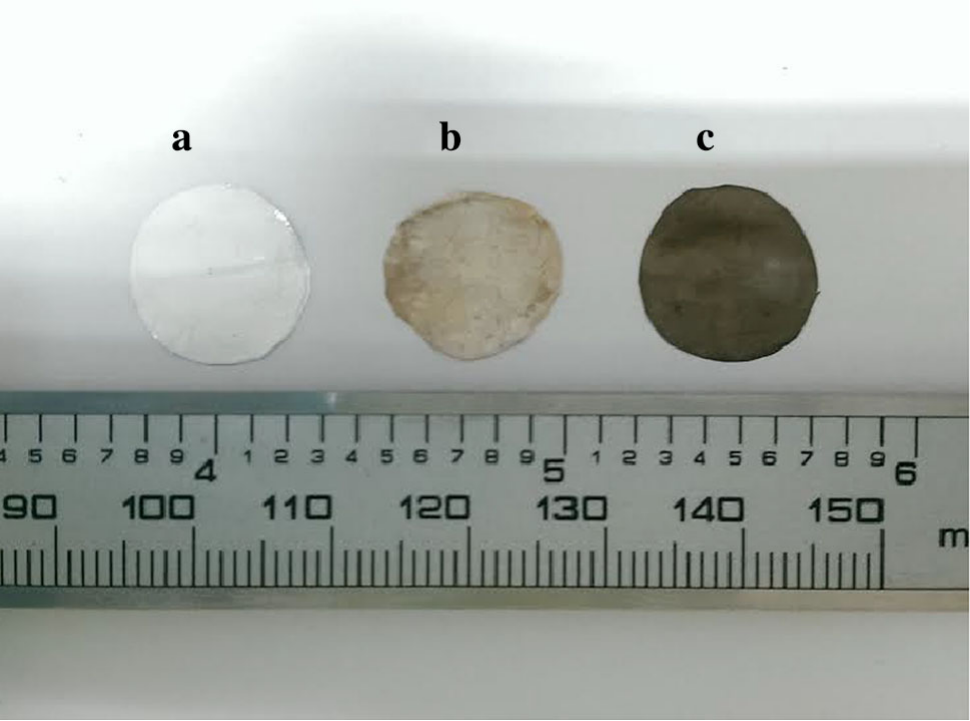
General appearance of the Ag-coated fibrous scaffold: **a** before coating with Ag (*white*) and **b**, **c** after photoreduction (**b** *light brown*, **c** *darker yellowish*-*brown*)

During the immersion of nanofibrous scaffolds in aqueous solution of AgNO_3_, PCL scaffold could not be fully immersed in the AgNO_3_ aqueous solution like the PCL/Ge (70:30) scaffold as PCL scaffold was hydrophobic. Both scaffolds changed colour gradually from white to light yellow after immersion in the AgNO_3_ solution, then to light brown after photoreduction. After photoreduction, scaffolds were placed in a Petri dish at room temperature. The scaffolds had shrunk slightly from the original dimensions and slowly changed colour to darker yellowish-brown after a few days. These changes are shown in Fig. 3.

It was observed that, without photoreduction, the colour of the scaffolds still changed to a darker yellowish-brown but required a longer period of time. It can be concluded that Ag^+^ ions can be easily reduced to Ag at room temperature and deposited on scaffolds, but the reduction process can be sped up by photoreduction using UV radiation. These scaffolds were characterized for Ag distribution, weight percentage, and chemical bonding.

#### Characterization

##### EDX mapping and EDX spectra of silver

**Fig. 4:**
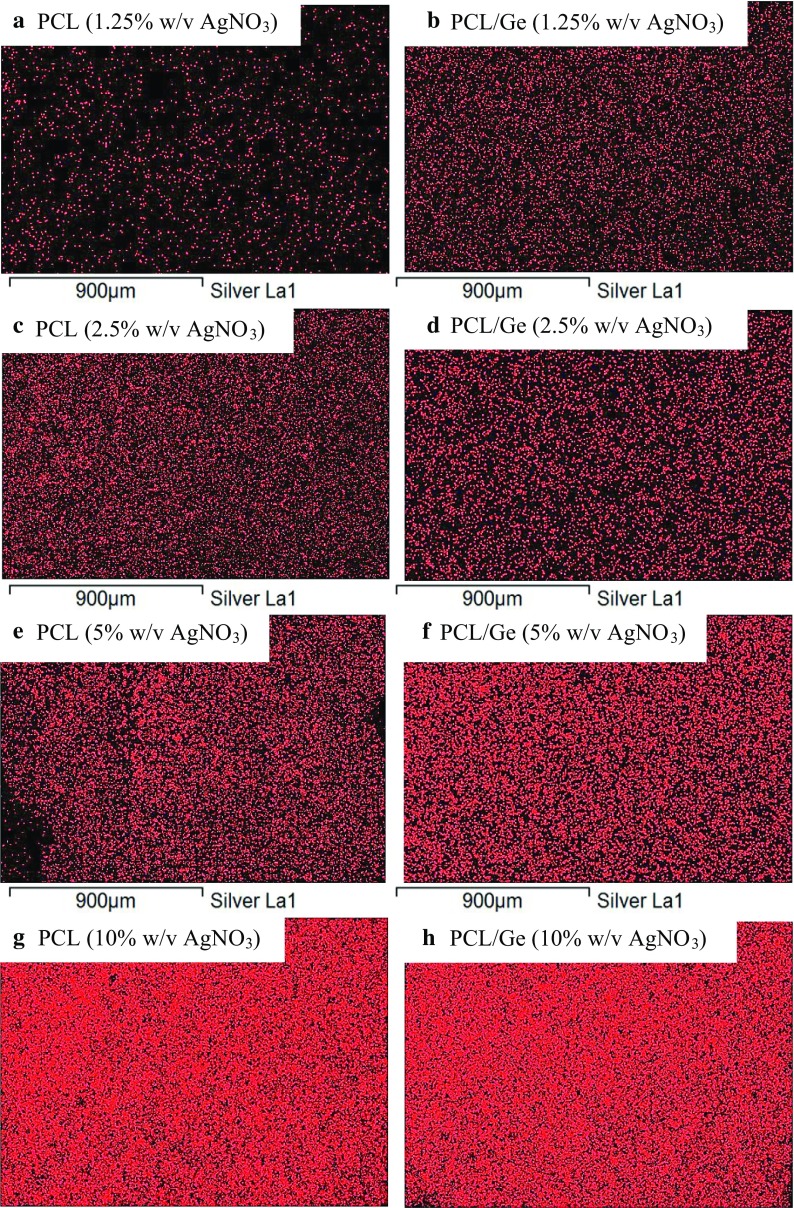
Ag distribution of PCL (*left*) and PCL/Ge (70:30) (*right*) nanofibrous scaffolds dipped in **a**, **b** 1.25 % w/v AgNO_3_; **c**, **d** 2.5 % w/v AgNO_3_; **e**, **f** 5 % w/v AgNO_3_; **g**, **h** 10 % w/v AgNO_3_

**Fig. 5:**
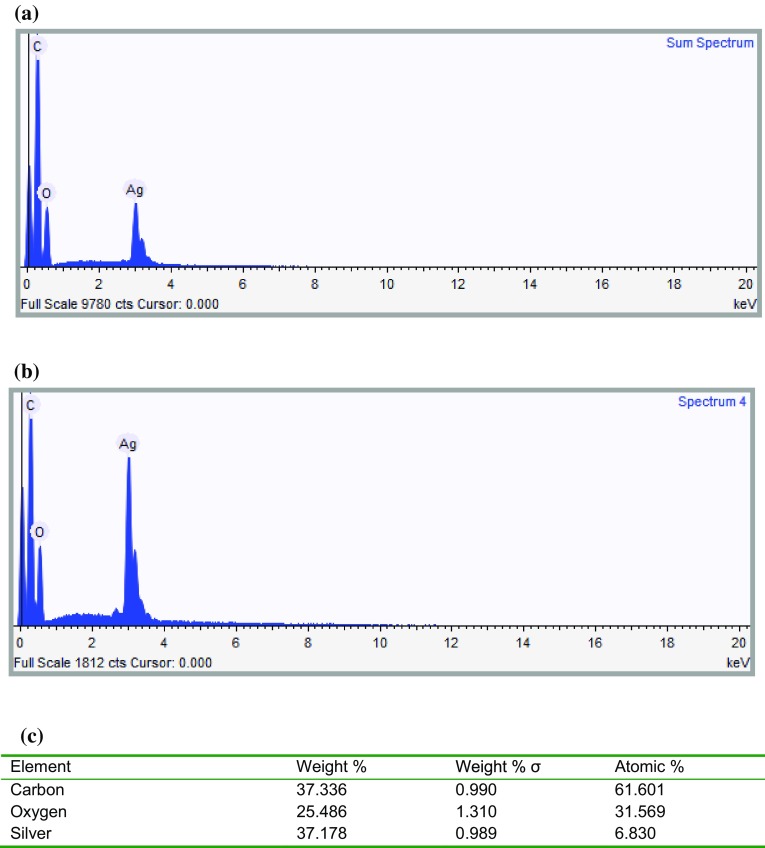
EDX of Ag-coated nanofibrous scaffold: **a** Ag/PCL, **b** Ag/PCL/Ge (70:30) and **c** elemental analysis of Ag-coated Ag/PCL/Ge nanofibrous scaffold

For the confirmation of Ag particles formation, scaffolds were characterized for Ag distribution using EDX. Through EDX mapping, the silver distribution on the nanofibrous scaffolds was determined. Figure 4 shows the EDX mapping and Fig. 5 shows the EDX spectra of Ag-coated PCL (Fig. 5a) and PCL/Ge (70:30) (Fig. 5b) and elemental analysis (Fig. 5c) of nanofibrous scaffolds. In Fig. 4, silver is represented by red dots. These results showed that Ag particles were formed. Ag peak of the EDX spectra in Fig. 5a, b and elemental analysis (Fig. 5c) confirmed the formation of Ag particles in the scaffolds.

There was a concern regarding the silver distribution on the PCL nanofibrous scaffolds as the hydrophobic scaffolds could not be fully immersed in the aqueous solution. Unexpectedly, the results show that the Ag distribution on both the PCL and PCL/Ge (70:30) nanofibrous scaffolds was homogeneous. With an increasing concentration of the AgNO_3_ solution, the weight percentage of Ag increased. However, the weight percentage of Ag on all PCL nanofibrous scaffolds was lower than on the PCL/Ge (70:30) nanofibrous scaffold because the PCL/Ge (70:30) nanofibrous scaffold was more hydrophilic.

##### Chemical bonding

**Fig. 6:**
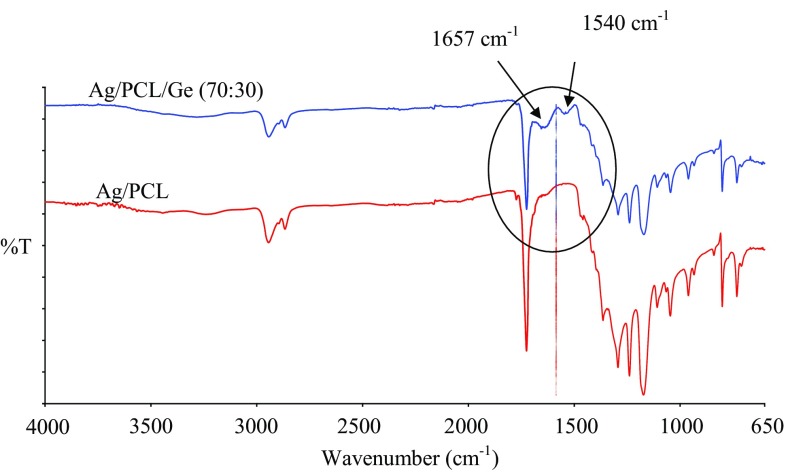
ATR spectra of Ag-coated PCL and PCL/Ge (70:30) nanofibrous scaffolds

Chemical bonding on the Ag-coated PCL and PCL/Ge (70:30) nanofibrous scaffolds was analysed using ATR spectra, as shown in Fig. 6. Comparing the ATR spectra of the PCL and PCL/Ge (70:30) nanofibrous scaffolds, two different bands were observed in the ATR spectrum of the PCL/Ge (70:30) nanofibrous scaffold, i.e., amide I at 1657 cm^−1^ and amide II at 1540 cm^−1^. The band at 1657 cm^−1^ represents carbonyl (C=O) stretching vibrations of the amide groups of Ge and the band at 1540 cm^−1^ represents the stretching of C–N bonds and bending of N–H bonds (Chong et al. 2015; Oraby et al. 2013). In Fig. 4, there was no shifting or additional bonding with Ag. Ag was not bonded to either the carbonyl groups of PCL or the amide groups of Ge. In conclusion, Ag was simply physically bonded to the PCL and PCL/Ge (70:30) nanofibrous scaffolds. Physically bonded Ag can be released easily from scaffolds to kill bacteria in a wound.

Antibacterial agents are needed especially at the primary stage to kill existing bacteria on a wound. As the pore size of nanofibres is relatively much smaller than the size of a bacterium, nanofibres can have sieve effect to block the penetration of bacteria and effectively prevent exogenous infection (Liu et al. 2010).

#### Ag^+^ ion release study

The antibacterial property of a scaffold depends on the role of Ag on the scaffold and its release behaviour. Table 1 shows the accumulated Ag^+^ ion release from the Ag/PCL and Ag/PCL/Ge (70:30) nanofibrous scaffolds after incubation in PBS for 1, 3, and 7 days. The amount of Ag^+^ ion release increased gradually until day 3 and decreased on day 7. Ag was physically attached to the PCL and PCL/Ge (70:30) nanofibrous scaffolds. Hence, Ag^+^ ions could be easily released from the nanofibrous scaffolds. A cell cytotoxicity study was performed to evaluate the toxicity of these scaffolds towards human cells. During the incubation process, Ag was converted into Ag^+^ ions and released from the nanofibrous scaffolds. Ag^+^ ions were released rapidly at the beginning due to high surface area of nanofibrous scaffolds. This result is similar to the findings of other studies (Depan et al. 2011; Xu et al. 2006). It can be suggested that the Ag/PCL and Ag/PCL/Ge (70:30) nanofibrous scaffolds show antibacterial properties. The antibacterial evaluation is discussed in the next section.

**Table 1:** Accumulative Ag^+^ ions release from Ag/PCL and Ag/PCL/Ge (70:30) nanofibrous scaffolds after incubation in PBS for 1, 3, and 7 days (*n* = 3)

Day	Ag^+^ ions release (ppm or mg/L)			
	Ag/PCL		Ag/PCL/Ge (70:30)	
	5 % AgNO_3_	10 % AgNO_3_	5 % AgNO_3_	10 % AgNO_3_
1	0.352 ± 0.004	0.390 ± 0.002	0.357 ± 0.006	0.442 ± 0.009
3	0.393 ± 0.020	0.467 ± 0.003	0.391 ± 0.020	0.492 ± 0.010
7	0.365 ± 0.040	0.402 ± 0.010	0.408 ± 0.005	0.462 ± 0.007

#### Antibacterial evaluation

The antibacterial effect of Ag-coated PCL and PCL/Ge (70:30) nanofibrous scaffolds was examined regarding their antibacterial activities against two types of bacteria that cause skin and wound infections, i.e., Gram-positive *B. cereus* (Bottone 2010; John et al. 2012) and Gram-negative *E. coli* (Rennie et al. 2000; Church et al. 2006). The zones of inhibition for Ag discs, PCL, PCL/Ge (70:30), Ag/PCL, and Ag/PCL/Ge (70:30) nanofibrous scaffolds after incubation were recorded. Table 2 shows the inhibition zones at 4, 24, and 48 h. The inhibition zone was observed as a clear circular area surrounding the nanofibrous scaffolds indicating the zone of bacteria that was killed or prevented from growing.

**Table 2:**
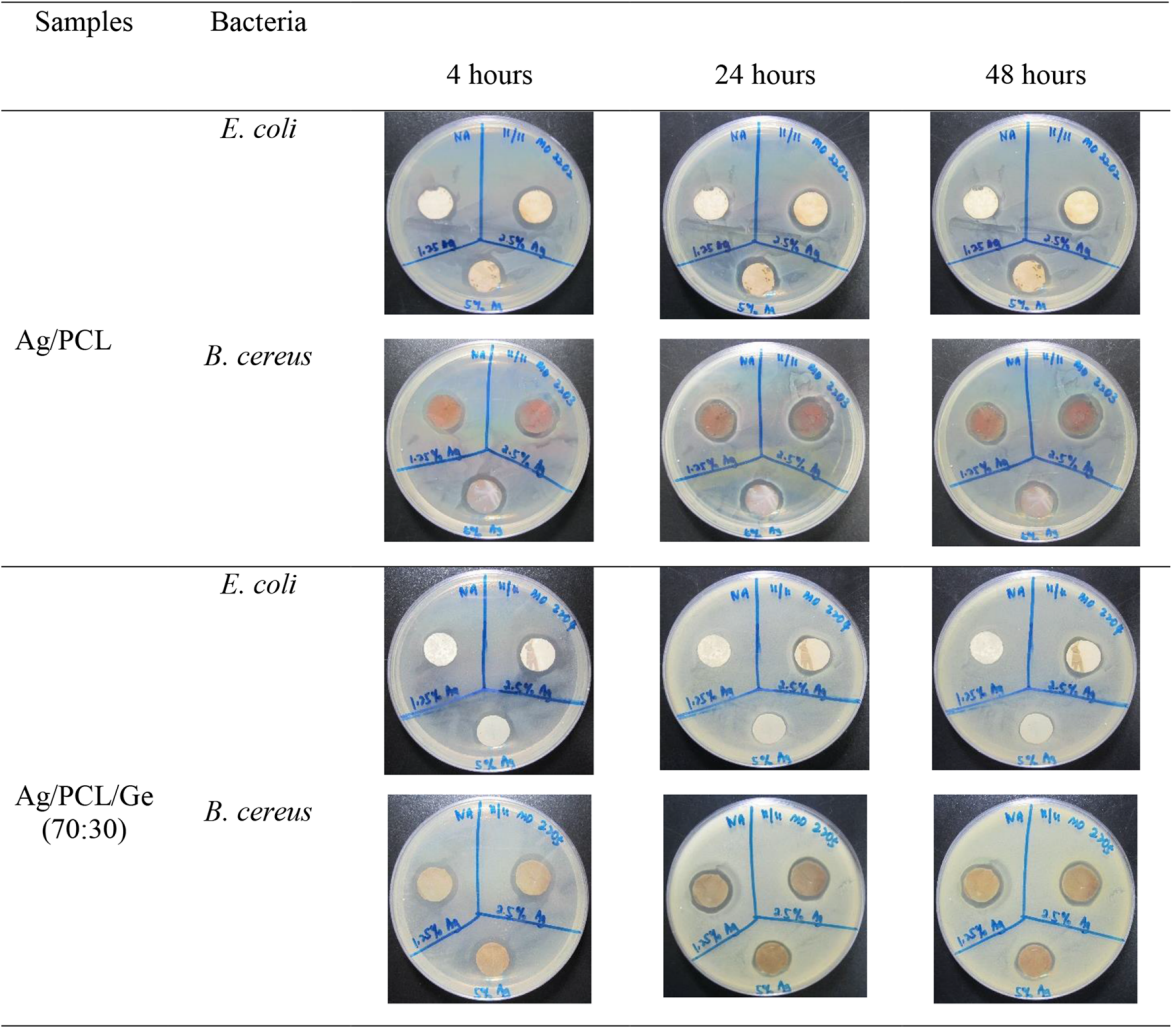
Antibacterial activity of PCL and PCL/Ge (70:30) at 4, 24, and 48 h

The inhibition zone of the control (Ag discs) showed an excellent antibacterial effect. The antibacterial effect was greater towards *E. coli* compared to *B. cereus*. According to the results in Table 2, the PCL and PCL/Ge (70:30) nanofibrous scaffolds did not have antibacterial properties as they showed no activity against either bacterium. However, according to Table 2, there was a clear inhibition zone around Ag-coated nanofibrous scaffolds for both bacteria, except for the Ag-coated PCL nanofibrous scaffold coated with the 1.25 % AgNO_3_ solution. This sample contained only 0.8 % Ag. This low concentration of Ag was not enough to kill the bacteria investigated. With an increase in the Ag content to 4.2 %, antibacterial properties were observed. There was no inhibition zone in the first and second hour as it takes time for Ag on the nanofibrous scaffolds to be ionized into Ag^+^ ions. After 4 h, an inhibition zone was seen, and the diameter of the inhibition zone was similar out to 48 h for all Ag/PCL and Ag/PCL/Ge (70:30) nanofibrous scaffolds.

Dipping of nanofibrous scaffolds into an AgNO_3_ aqueous solution is a simple and useful method to endow scaffolds with antibacterial properties. These results have shown that Ag exerts a strong antibacterial effect. Ag^+^ ions are bioactive and, in sufficient concentrations, kill bacteria effectively as shown by the inhibition zone in Table 2.

#### Cell cytotoxicity using the MTT assay

**Fig. 7:**
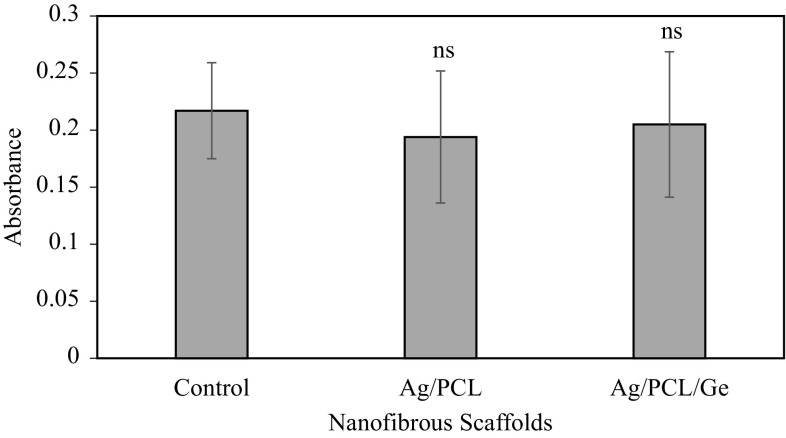
Cell cytotoxicity on control, Ag-coated PCL and PCL/Ge (70:30) nanofibrous scaffolds. Data are representative of three independent experiments and are plotted as mean ± SD (*n* = 3). ns, not significant

Ag-coated PCL and PCL/Ge (70:30) nanofibrous scaffolds were further investigated for cell cytotoxicity using the MTT assay. The cell cytotoxicity results are shown in Fig. 7. Both the PCL and PCL/Ge (70:30) nanofibrous scaffolds showed high absorbance, which was similar to the control; these data were not significantly different. This indicated that both of these scaffolds were non-toxic to cells.

It has been reported that an Ag-coated polyvinyl alcohol (PVA) nanofibrous scaffold coated with a 5 % w/v AgNO_3_ aqueous solution was non-toxic to cells and showed good antibacterial activity; it also promoted wound healing in vivo in female Sprague–Dawley rats (Liu et al. 2010). Since Ag-coated scaffolds using a 5 % w/v AgNO_3_ solution showed no cytotoxicity, it can be concluded that a concentration lower than a 5 % w/v AgNO_3_ solution would be non-toxic as well. Therefore, in this study, only the PCL and PCL/Ge (70:30) nanofibrous scaffolds coated with the 10 % w/v AgNO_3_ aqueous solution were investigated and were found to be non-toxic to cells.

## Conclusions

Ag was coated effectively on PCL and PCL/Ge nanofibrous scaffolds by using dipping method in AgNO_3_ aqueous solution followed by photoreduction. Ag was physically bonded to the scaffolds and was non-toxic to cells. Ag concentration of at least 4.2 % was sufficient to exert antibacterial properties against *B. cereus* and *E. coli*. As average fibre diameter of the PCL/Ge (70:30) nanofibrous scaffold was in the range of the collagen fibre diameter in ECM, and should provide a similar environment for cell growth as that found in the body. Hence, the Ag/PCL/Ge (70:30) nanofibrous scaffold has excellent potential to be used as a promising scaffold with antibacterial properties for tissue engineering applications to prevent bacterial infection and promote tissue regeneration.
